# Analysis of the Influence of Quantile Regression Model on Mainland Tourists' Service Satisfaction Performance

**DOI:** 10.1155/2014/763573

**Published:** 2014-01-16

**Authors:** Wen-Cheng Wang, Wen-Chien Cho, Yin-Jen Chen

**Affiliations:** ^1^Department of Business Administration, National Taiwan University of Science and Technology, Taiwan; ^2^Department of Business Administration, Hwa Hsia Institute of Technology, No. 111 Gongzhuan Road, Zhonghe District, New Taipei City 235, Taiwan; ^3^Department of Hospitality Management, Hsing Wu University, No. 101, Sec. 1, Fenliao Road, LinKou District, New Taipei City 24452, Taiwan; ^4^Department of Hotel and Restaurant Management, Ching Kuo Institute of Management and Health, 336 Fu Hsin Road, Keelung, Taiwan

## Abstract

It is estimated that mainland Chinese tourists travelling to Taiwan can bring annual revenues of 400 billion NTD to the Taiwan economy. Thus, how the Taiwanese Government formulates relevant measures to satisfy both sides is the focus of most concern. Taiwan must improve the facilities and service quality of its tourism industry so as to attract more mainland tourists. This paper conducted a questionnaire survey of mainland tourists and used grey relational analysis in grey mathematics to analyze the satisfaction performance of all satisfaction question items. The first eight satisfaction items were used as independent variables, and the overall satisfaction performance was used as a dependent variable for quantile regression model analysis to discuss the relationship between the dependent variable under different quantiles and independent variables. Finally, this study further discussed the predictive accuracy of the least mean regression model and each quantile regression model, as a reference for research personnel. The analysis results showed that other variables could also affect the overall satisfaction performance of mainland tourists, in addition to occupation and age. The overall predictive accuracy of quantile regression model *Q*0.25 was higher than that of the other three models.

## 1. Introduction 

The opening up of Taiwan to visitors from mainland China can boost Taiwan's tourism industry and periphery industries. Based on the estimate by the Taiwan Visitors Association, if Taiwan opens up to 3,000 mainland visitors per day, there will be 1 million visitors per year. If each mainland visitor stays in Taiwan for seven to ten days and spends 50,000 NTD, this could contribute about 50 billion NTD to Taiwan's tourism industry. Due to the multiplier effect of consumption, the output value of the Taiwan service industry could reach over 100 billion NTD, in industries such as the airline industry, travel industry, tourist hotels, transportation, food, recreation areas, department stores, and native products. This represents good news, and it can increase the number of employment opportunities in Taiwan. However, the business opportunity resulted from opening up to mainland visitors is too large for Taiwan. The aim of this paper was to discuss whether Taiwan has engaged in appropriate planning and relevant industry software and hardware facilities and services.

A great deal of the literature on consumer behavior already exists [1, 2]; however, the literature on mainland visitors traveling to Taiwan is seldom found. Thus, the question items for the satisfaction of mainland visitors with Taiwan's tourism services were used as the research basis in this study. This paper conducted a questionnaire survey of mainland tourists. The questionnaire included basic data and nine satisfaction items. First, grey relational analysis was conducted on the nine question items of satisfaction, and the grey relational grade of the analysis result was regarded as the overall satisfaction performance value through the overall satisfaction performance could be learned. Next, the basic data and the first eight satisfaction items were used as the independent variables (*X*), and the overall satisfaction performance was used as the dependent variable (*Y*). The least mean square regression model and quantile regression model analysis were used for analysis. The difference between the two models was compared, and the relationships among the independent variables and the dependent variable were observed under different quantiles. At last, this study discussed the predictive accuracy of the least mean regression model and each quantile regression model, and the results could be provided as a reference for research personnel.

This paper is organized as follows.  Section 1 presents the motivation and purpose of the research.  Section 2 introduces grey correlation performance analysis, the quantile regression model, and relevant literature.  Section 3 involves the sample data and empirical analysis.  Section 4 proposes the research conclusion.

## 2. Methodology 

### 2.1. Grey Mathematics and Grey Relational Analysis

The grey mathematic theory, proposed by Long [3], is used under a situation of system uncertainty and information incompleteness to perform the model setup, relational analysis, and forecasts, so as to understand the system. Before performing grey relational analysis, in order to let the sequence satisfy comparability requirements, a normalization treatment needs to be performed on the data of the sequence. This is called the formation of the grey relation, in which the sequence can be made to satisfy comparability. Before performing grey relational analysis, the reference sequence has to be confirmed first, and then the closeness between other sequences (comparison sequences and son sequences) and the reference sequence can be compared so as to find out the level of grey relation and create the grey relation ordinals. Through the rankings, the advantages and disadvantages can be judged so as to assist in carrying out the decision. The steps of grey relational analysis are as follows.

(A) From the original decision matrix *D*, find out standard sequence *A*_0_ and inspected sequence *A*_*i*_. The standard sequence is set *A*_0_ = (*x*_01_, *x*_02_,…, *x*_0*j*_,…, *x*_0*n*_) formed by the ideal target value of each influencing factor and has a total of *j* terms, wherein *j* = 1, 2, …, *n*. In addition, the performance value of inspected sequence *A*_*i*_ = (*x*_*i*1_, *x*_*i*2_,…, *x*_*ij*_,…, *x*_*in*_), wherein *i* = 1, 2, …, *m*.
(B) Normalize the data of original decision matrix *D*.
(C) Calculate grey relational distance Δ_0*ij*_ and use it to evaluate the difference between each normalized value and normalized reference data:
  (1) Δ0ij=|x0j∗−xij∗|.
(D) Calculate grey relational coefficient *γ*_0*ij*_:
  (2) γ0ij=Δmin+ζΔmaxΔ0ij+ζΔmax.
(E) Calculate grey relational grade Γ_0*i*_ by the following equation:
  (3) Γ0i=∑j=1n[ωj×γ0ij].
  Rank the grey relational ordinal and follow grey relational grade value to perform performance ranking.

Grey relational analysis has been applied in many industries, such as business [4], education [5], hospitals [6], and tourism [7]. In this paper, grey relational analysis was performed to discuss the satisfaction of visitors from mainland China traveling to Taiwan.

### 2.2. Quantile Regression Method

The quantile regression method [8] is an extension of the traditional least square regression method. The difference is that the former can accurately estimate the marginal effect of independent variables on the dependent variable in a specific “condition” quantile, which is superior to the marginal effect of the latter's average trend. The quantile regression method has one more observation dimension than that of the traditional least square regression, and it can therefore analyze and observe the margin effect of each specific quantile condition. Quantile regression has been applied to financial research [9–11]. In this study, it is applied to the tourism industry.

**Figure 1:**
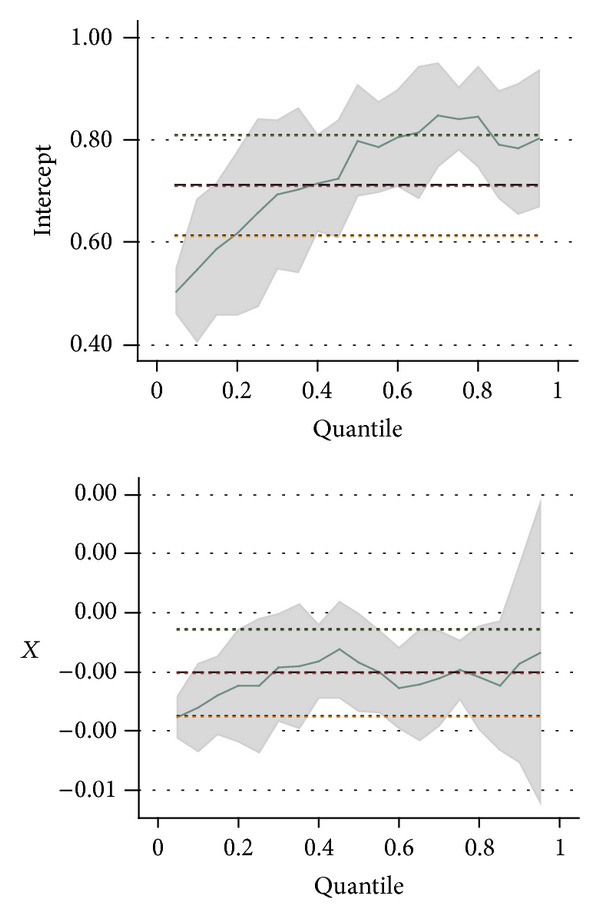
Quantile regression diagram.

STATA was used to perform quantile regression analysis; its graphics is similar to Figure 1. It was based on the average concept of the traditional least square method but was extended to different quantile positions of the entire distribution interval. From this, the impact of the basic data of the questionnaires and the first eight satisfaction items (*X*) on the overall satisfaction and the impact of the ninth question item (*Y*) were observed.

## 3. Empirical Research

### 3.1. Sample Data and Variables

There were a total of 144 mainland visitors traveling to Taiwan who participated in the questionnaire survey. The question items in the questionnaires were written using simplified Chinese characters and included basic information items such as gender (*A*_1_), age (*A*_2_), occupation (*A*_3_), and favorite Taiwan food type (*A*_4_). The satisfaction question items included Taiwan tourist attractions (*X*_1_), Taiwanese food (*X*_2_), Taiwanese hotel facilities (*X*_3_), Taiwanese hotel service attitude (*X*_4_), Taiwanese night fair culture (*X*_5_), Taiwanese architectural styles (*X*_6_), Taiwan weather conditions (*X*_7_), Taiwanese road cleanliness (*X*_8_), and whether to visit Taiwan in the next time (*X*_9_). The descriptive statistical values of these satisfaction question items are shown in Table 1.

### 3.2. Quality Satisfaction Analysis

**Figure 2:**
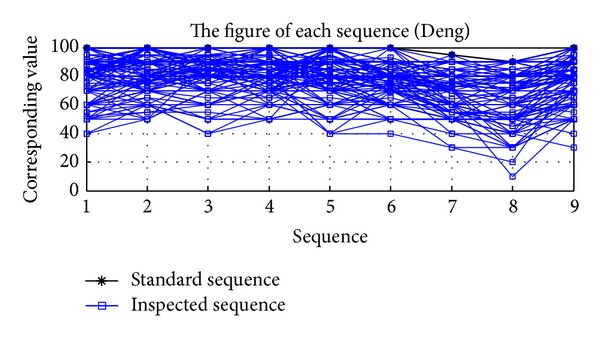
Output chart of grey relational grade of eight satisfaction items.

This paper used the grey relational grade analysis proposed by Deng and the grey relational analysis MATLAB procedure developed by Wen et al. [12] to calculate the grey relational grade and then analyze the satisfaction performance of the nine question items. Higher service satisfaction values of the nine question items are better. In this paper, the maximum value of the nine question items was used as the standard sequence. The grey relational analysis is shown in Figure 2, in which the upper bold black line denotes the standard sequence and the thin solid line denotes the inspected sequence. Each piece of data included nine nodes which denoted the service satisfaction values of the nine question items. If the inspected sequence was closer to the standard sequence, the service quality satisfaction in the questionnaire information would be better; namely, the customers would be more satisfied with the contents in the nine question items. The grey relational analysis result indicated the three respondents to evaluate the overall satisfaction; the highest were the 97th respondent (GRG = 0.921), the 65th respondent (GRG = 0.8988), and the 125th respondent (GRG = 0.8864). The gray relational grade in the analysis result was regarded as the overall satisfaction performance value, namely, the dependent variable (*Y*). In conjunction with the first eight question items (*X*), the least square regression model and quantile regression were used for analysis.

### 3.3. The Past Eight Satisfaction Items Are Used as the Independent Variables; the Overall Satisfaction Performance Is Used as One Dependent Variable for Regression Model Analysis

**Figure 3:**
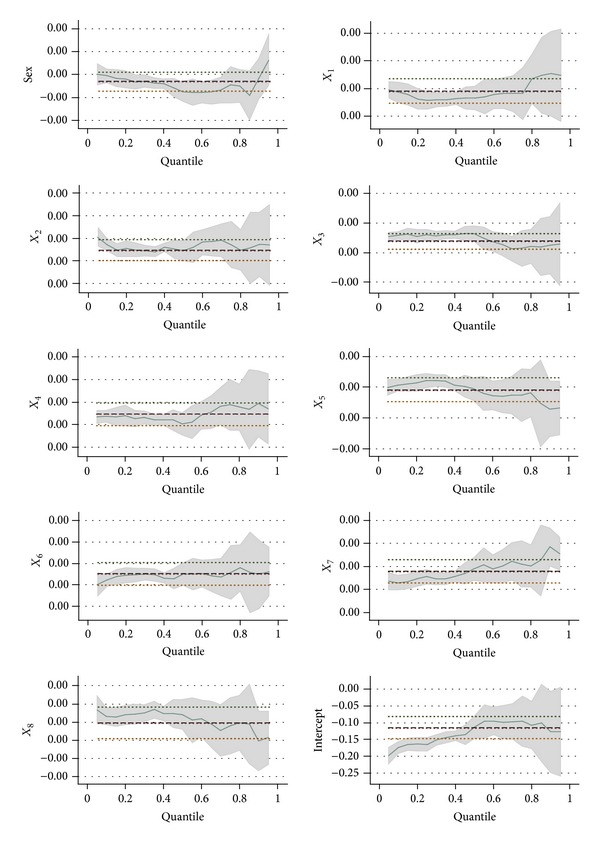
Output of analysis results of least square regression model and quantile regression model for overall satisfaction performance.

In this paper, the first eight satisfaction items were used as independent variables (*X*) and the gray relational analysis result was used as the dependent variable (*Y*). The least square regression model and quantile regression model were used for analysis. The quantile regression model was established using the 0.25 quantile (1/4 quantile), 0.5 quantile (1/2 quantile), and 0.75 quantile (3/4 quantile). The analysis results are shown in Table 2 and Figure 3.

As shown in Tables 2 and 3, gender had an insignificant impact on the overall satisfaction performance in the high quantiles, but it had a significant impact on the overall satisfaction performance in the low quantiles (0.25 and 0.5). In Figure 3, the long thick dotted line denotes the least regression model, and the upper and lower short fine dotted lines denote the confidence interval. From Figure 3, it could be seen that the quantile regression overestimated the situation under the 0.25 quantile and that the quantile regression underestimated the situation under the 0.25 quantile–0.5 quantile, as compared to the least square regression model. The questionnaires showed that the overall satisfaction of men was lower than that of women, because Taiwanese hotels pay more attention to women. The hotels failed to provide smoking areas for male guests, and they were not allowed to speak loudly or drop litter and cigarette ends. The male guests thus felt inconvenienced, and their overall satisfaction was lower. Taiwan tourism enterprises and relevant governmental department should work to solve these problems.

From Table 2, it could be found that variable *X*_1_, satisfaction with tourist attractions, reached the significance level. Thus, the satisfaction of Taiwanese tourist attractions could significantly affect the overall satisfaction performance. As shown in Figure 3, if the satisfaction with tourist attractions was estimated using the least square regression model, the high quantile could be underestimated. This revealed that mainland visitors who had high overall satisfaction performance also had higher requirements for tourist attractions. Namely, Taiwanese tourist attractions could affect the evaluation of Taiwan's overall tourism quality by mainland visitors. In Taiwan, some famous scenic spots have complete management planning, such as Ali Mountain and Sun Moon Lake; however, other less-famous scenic spots often trigger complaints due to the lack of management planning. Thus, the management of Taiwanese tourist attractions should be enhanced, with the help of relevant government agencies.

From Table 2, it could be found that satisfaction with food reached the significance level under different quantiles. Thus, satisfaction with food could also affect the overall satisfaction performance. From Figure 3, if the least square regression model was used for estimation, the overall satisfaction would be underestimated under different quantiles. This indicated that Taiwanese food was an important indicator to measure whether the mainland tourists were willing to visit Taiwan, regardless of low or high overall satisfaction. Thus, relevant departments should give assistance to enterprises in the food industry. Taiwanese food has unique features and can attract more visitors, thus increasing the inbound tourist flow.

It was found that hotel facilities (*X*_3_) were not significant under the 0.75 quantile, as shown in Tables 2 and 3. If the least square regression model was used, this variable would be underestimated under the low quantiles. This revealed that the worse overall satisfaction performance of the visitors was caused by poor hotel facilities. Hotel service attitude (*X*_4_) reached the significance level under different quantiles; however, if the least square regression model was used, the variable could be overestimated under the low quantiles and be underestimated in the high quantiles. This revealed that the requirements for hotel service attitude would become higher and higher with the improvement of the overall satisfaction performance of mainland tourists. Thus, Taiwan's relevant departments should give assistance to hotels in the improvement of hotel facilities and staff service attitudes.

It was found that night fair culture, architectural styles, and weather conditions (*X*_5_, *X*_6_, and *X*_7_) reached the significance level, as shown in Table 2. Taiwan has many night fairs throughout the island, and many tourists travel to Taiwan in order to taste local food at the night fairs. Taiwan also has many famous scenic spots and historical sites, such as Fort Santo Domingo in New Taipei City, Taipei's National Palace Museum, and Fort Provintia and Fort Zeelandia in Tainan. The architectural style of many famous temples is also an important factor that attracts foreign tourists. In addition, Taiwan is located in the subtropical zone, and the weather is spring-like all year round, which can attract more tourists to come to Taiwan.

Tables 2 and 3 show that street cleanliness (*X*_8_) had a significant difference under the high and low quantiles. It did not reach the significance level under the 0.75 quantile, but it reached the significance level under the 0.25 quantile. Thus, street cleanliness was an important indicator for the mainland tourists with lower satisfaction performance. In Taiwan, street sweepers are responsible for cleaning rubbish on the street, and such rubbish is often caused by road construction. This problem should be solved by the urban development bureaus. In addition, some parts of the sidewalks are illegally occupied. These problems could result in the worse satisfaction performance of mainland tourists. The Taiwanese tourist department should coordinate with the relevant government departments for road improvement.

### 3.4. Division of Sample Data

In this section, the quantile regression model was used to analyse the reliability of the satisfaction of mainland tourists and determine the accuracy of the quantile regression model. The original sample data were divided into four groups; three groups were used as training data to establish the regression model, and one group was used as the testing data for cross-validation of the prediction methods, so as to test the reliability and predictive ability of the models. In this paper, five assessment indicators and the overall predictive accuracy were used to compare the predictive ability of the four models, as follows:

(1) the root mean squared error (RMSE):
  (4) RMSE=∑t=1nxt−x^tN,
(2) the revised Theil inequality coefficient (RTIC):
  (5) RTIC=[∑t=1N(Xt−X^t)2∑t=1Nxt2]1/2,
(3) the mean absolute error (MAE):
  (6) MAE=1M∑l=1M|Zt+1−Z^t(l)|,
  where *M* is the number of predictive values, *Z*_*t*+1_ is observed value of one hour, and ${\hat{Z}}_{t}(l)$ is estimated value of one hour,
(4) the mean absolute percentage error (MAPE):
  (7) MAPE=(1M∑l=1M|(Zt+1−Z^t(l))Zt+1|)×100%,
(5) the coefficient of efficiency (CE):
  (8) CE=1−∑(xt−x^t)2∑(xt−x−t)2.

For indicators 1 to 4, a result closer to 0 indicated that the model had higher accuracy. For the fifth indicator, a result closer to 1 indicated that the model had higher accuracy. The analysis and test results are shown in Table 2.

Table 4 shows that, in the *Q*0.25 predictive model, the RMSE was 1.024, the RTIC was 0.019, the MAE was 0.996, and the MAPE was 0.011, which were lower than the results for OLS in *Q*0.5 and *Q*0.75. The CE was 0.967 and the overall accuracy was 97.6%, which was higher than that of the other three models. Thus, the *Q*0.25 predictive model had better predictive ability as compared to the other three models. The analysis results showed that the overall predictive accuracy of the three quantile regression models was almost 90%. In terms of the pseudo *R* ^2^ in Table 2, the regression models had high interpretability; thus, the quantile regression had a high confidence level when it was used to analyze the impact of the variables on the overall satisfaction of mainland tourists.

## 4. Conclusion

The main contribution of this paper is different from the past literature. In this paper, quantile regression and the OLS method were used to discuss the impact of the satisfaction of mainland tourists with service quality on the overall satisfaction performance. The causes for the impact of the variables on the overall satisfaction under different quantiles and improvement solutions were further analyzed. The empirical results showed that gender, tourist attractions, hotel facilities, night fair culture, and street cleanliness affected the overall satisfaction performance of the mainland tourists and had significant differences in the high and low quantiles. Besides work and age, other variables could also affect the overall satisfaction performance of mainland tourists. In addition, quantile regression was used to analyze the reliability of the satisfaction of mainland tourists in this paper. Further, the original data were divided into several groups to establish the prediction models. Five evaluation indicators and the overall predictive accuracy were used to conduct cross verification and analysis of the four models. The findings showed that the overall accuracy of the *Q*0.25 quantile regression predictive model reached a level of 97.6% and that *Q*0.5 and *Q*0.75 reached the 90% level. Thus, the quantile regression model is feasible for analyzing the impact of variables on the satisfaction of mainland tourists traveling to Taiwan.

## Figures and Tables

**Table 1:** Descriptive statistic value of the nine satisfaction variables.

Satisfaction	*X*_1_	*X*_2_	*X*_3_	*X*_4_	*X*_5_	*X*_6_	*X*_7_	*X*_8_	*X*_9_
Max	100	100	100	100	100	100	95	90	100
Min	40	50	40	50	40	40	30	10	30
Avg	80.76	83.26	81.17	81.88	79.84	76.24	70.38	67.19	78.10
Std	14.15	12.74	12.10	11.80	13.90	11.57	13.30	16.80	13.86
*N*	144	144	144	144	144	144	144	144	144

**Table 2:** Analysis results of least square regression model and quantile regression model for overall satisfaction performance.

Regression variable	OLS			*Q*0.25			*Q*0.5			*Q*0.75		
	Coefficient	*T* value	Significant	Coefficient	*T* value	Significant	Coefficient	*T* value	Significant	Coefficient	*T* value	Significant
Sex	−0.004	−1.074		−0.0001	−1.77	*	−0.0001	−2.93	***	−0.0001	−1.13	
Occupation	0.001	1.004		0.0013	0.43		−0.0025	−0.63		−0.0057	−0.90	
Age	−0.002	−0.949		−0.0001	−0.24		−0.0001	−0.08		0.0002	0.19	
Tourist attraction	0.001	3.837	***	0.0006	2.64	***	0.0007	2.45	**	0.0008	2.02	**
Food	0.001	6.103	***	0.0015	5.56	***	0.0015	4.18	***	0.0017	4.05	***
Hotel facilities	0.001	2.989	***	0.0012	4.60	***	0.0013	3.46	***	0.0003	0.52	
Hotel service attitude	0.002	6.016	***	0.0013	4.86	***	0.0010	2.84	***	0.0019	3.89	***
Night fair culture	0.001	4.552	***	0.0012	5.64	***	0.0009	3.40	***	0.0007	1.91	*
Architectural style	0.001	5.568	***	0.0015	5.44	***	0.0015	4.43	***	0.0016	3.16	***
Weather conditions	0.002	7.091	***	0.0015	5.33	***	0.0019	4.43	***	0.0021	5.39	***
Street cleanliness	0.000	2.167	**	0.0007	2.61	***	0.0007	2.20	**	0.0004	1.11	
Pseudo *R* ^2^	0.9670			0.8744			0.8349			0.7985		

Note: *is significant at the 10% significance level; **is significant at the 5% significance level; ***is significant at the 1% significance level.

**Table 3:** Verification result of coefficient difference under high and low quantiles (*Q*0.25–*Q*0.75).

Variable	Sex	Occupation	Age	Tourist attraction	Food	Hotel facilities	Hotel service attitude	Night fair culture	Architectural style	Weather conditions	Street cleanliness
*F* value	3.02	0.11	0.20	3.16	0.12	5.15	0.36	3.41	0.03	1.59	4.59
Different significance	*			*		***		**			***

Note: *is significant at the 10% significance level; **is significant at the 5% significance level; ***is significant at the 1% significance level.

**Table 4:** Evaluation indicators and result of total predictive accuracy.

Regression	RMSE	RTIC	MAE	MAPE	CE	Accuracy %
OLS	1.788	0.031	1.526	0.017	0.903	91.25
*Q*0.25	1.024	0.019	0.996	0.011	0.967	97.60
*Q*0.5	1.332	0.026	1.288	0.015	0.935	94.87
*Q*0.75	2.156	0.045	1.961	0.030	0.866	89.16
